# PP26 Systematic Literature Review Of Funding Models For The Access To Drugs For Patients With Rare Diseases

**DOI:** 10.1017/S0266462324001995

**Published:** 2025-01-07

**Authors:** Constanza Vargas Parada

## Abstract

**Introduction:**

There is no comprehensive framework that considers the various features of current funding models on drug accessibility for rare diseases; such a framework would assist policymakers to more effectively meet the challenges of these patients. This article reviews the funding models implemented worldwide to facilitate this access.

**Methods:**

The PRISMA guidelines were used to conduct a systematic literature review. The following databases were searched: Ovid (Embase/MEDLINE), Cochrane database, Web of Science, EconLit, the National Institute for Health and Care Research (NIHR), Centre for Review and Dissemination (CRD), and International Network of Agencies for Health Technology Assessment (INAHTA). Two independent reviewers screened all titles and abstracts, and one reviewer did the full-text review and data extraction. Data were collected on general study characteristics, general aspects of rare diseases, source of funding, allocation of resources, and pricing strategies.

**Results:**

A total of 3,815 unique citations were screened, and 148 were included for data extraction. Each funding model was characterized based on its unique features specific to rare diseases, focusing on process, methods applied, and consideration of attributes. Sixty funding models were identified in 41 countries, categorized as separate processes (42%), exceptions to standard processes (32%), standard processes with no changes (23%), and alternative pathways (3%). More than one funding model was available for 29 percent of countries. Funding models varied in their approach to HTA, source of funding, consideration of uncertainty, and pricing strategies.

**Conclusions:**

The diversity of funding models highlights the complexity of addressing access to treatments for rare diseases. Special considerations towards rare diseases generally targeted the greater uncertainty in the clinical evidence. Despite the existing platform that enables access for drugs for rare diseases, only 10 percent of rare diseases have an available treatment and fewer patients can access these technologies.

